# Making space for criminalistics: Hans Gross and *fin-de-siècle* CSI

**DOI:** 10.1016/j.shpsc.2012.09.002

**Published:** 2013-03

**Authors:** Ian Burney, Neil Pemberton

**Affiliations:** Centre for the History of Science, Technology and Medicine, University of Manchester, Manchester M13 9PL, UK

**Keywords:** Forensic science, Crime scene investigation, Trace evidence, Hans Gross, Criminalistics, Criminology

## Abstract

This article explores the articulation of a novel forensic object—the ‘crime scene’—and its corresponding expert—the investigating officer. Through a detailed engagement with the work of the late nineteenth-century Austrian jurist and criminalist Hans Gross, it analyses the dynamic and reflexive nature of this model of ‘CSI’, emphasising the material, physical, psychological and instrumental means through which the crime scene as a delineated space, and its investigator as a disciplined agent operating within it, jointly came into being. It has a further, historiographic, aim: to move away from the commonplace emphasis in histories of forensics on *fin-de-siècle* criminology and toward its comparatively under-explored contemporary, criminalistics. In so doing, it opens up new ways of thinking about the crime scene as a defining feature of our present-day forensic culture that recognise its historical contingency and the complex processes at work in its creation and development.

This article has two aims. One is historical: to explore the late-nineteenth century roots of what has become a defining feature of our present-day forensic culture—the crime scene as a distinct domain of investigation and analysis. We will do this through a detailed engagement with one historical actor, the Austrian jurist and magistrate Hans Gross, and one of his books, *Handbuch für untersuchungsrichter als system der kriminalistik* (1893, translated as *Criminal Investigation: A Practical Handbook* in 1906). The other, with which we begin, is historiographical: to use this account of ‘Grossian’ crime scene investigation to show the value of shifting attention away from the much studied case of *fin-de-siècle* criminology and onto its comparatively underexplored contemporary, criminalistics.

## 1 Separating criminology and criminalistics

In recent decades historians such as Neil Davie, Mary Gibson, Robert Nye, Daniel Pick and Richard Wetzell have provided sophisticated accounts of the disciplinary formation of criminology and its core object of concern, the criminal body. Criminology, in these accounts, was shaped by modes of thinking drawn from evolutionary biology, anthropology and anthropometrics, interlaced with degenerationist anxieties and fears of national decline, of which the Italian criminologist Lombroso has been taken as its emblematic practitioner.^1^ This scholarly interest in the congruity between criminology and culture, between ideas about the criminal body and those about the body social, though productive and important, has nonetheless come at a price. It has marginalised a contemporaneous forensic enterprise that, arguably, has greater relevance to the historical path that forensics followed over the next century—namely, the scientific investigation of the circumstances of a specific crime and the identification of a specific culprit as an end in itself (*criminalistics*), rather than mapping these as data points within one of the innumerable taxonomic subdivisions of contemporary criminology.^2^

Separating criminalistics from criminology, of course, does not deny the existence of historical and conceptual convergences between the two, but rather seeks out the fertile space that opens up once separation is achieved. Allan Sekula’s incisive analysis of the scientific, technical and administrative underpinnings of late nineteenth-century attempts to capture criminal identity provides a glimpse of what this approach might yield. Sekula shows that while the Parisian police bureaucrat Alphonse Bertillon and the English statistician and founder of eugenics Francis Galton worked within a shared cultural moment—and thereby engaged with a common set of questions (the control of criminality via mastery of the criminal body) and tools with which to achieve this (e.g. the camera and the calliper)—they nonetheless ‘constitute two methodological poles of the positivist attempts to define and regulate social deviance.’^3^ For Galton, the holy grail was unlocking the secrets of race, inheritance and degeneration via the measurement and statistical analysis of bodily characteristics; for Bertillon, it was the use of these characteristics to link an individual body to a record of an individual malefactor already entered into the police files. Galton’s search led to the composite photograph, Bertillon’s to the *portrait parlé*.

Simon Cole’s excellent history of criminal identification extends this point by showing how both Bertillion’s anthropometric system and its ultimately more successful rival, fingerprinting, were conceptually and technically positioned at the cusp of the criminological/criminalistic divide. Bertillon (son of an eminent demographer, anthropologist and Quetelet disciple; police clerk in the eye of France’s recidivist storm) devised his system to solve the problem of individual identity, but did so in answer to an agenda set in large part by criminological concerns, and with reference to objects (e.g. ears, tattoos) and tools (again, calliper and camera) that were of shared currency. Little wonder that Lombroso welcomed Bertillion as a fellow traveller. Fingerprinting was an equally hybrid product: in its modern incarnation it emerged from within an imperial judicial apparatus focused on containing native ‘criminal castes’. For many of its most influential early proponents fingerprints linked to this project by serving not as marks of individual differentiation but as markers of racial, ethnic and characterological groupings. The fact that this latter version of fingerprinting is now largely forgotten is, for Cole, the outcome of a concerted effort on the part of subsequent fingerprint examiners, who were seeking to establish themselves as experts worthy of trust, to disassociate themselves from speculative over-reaching: ‘By turning the fingerprint into an empty signifier—a sign devoid of information about a body’s race, ethnicity, heredity, character, or criminal propensity—fingerprint examiners made fingerprint identification seem less value-laden, more factual.’^4^

The ‘selective amnesia’^5^ that enabled fingerprinting, by erasing its criminological twin, to emerge as a straightforward tool of criminalistics, also operates in the other direction: that is, the interpretive allure of the (ultimately dead-end) problematic of ‘criminal types’ has worked against an appreciation of the range of contemporaneously developing criminalistic practices that extended beyond those associated with the problem of individuation. Breaking this latter historiographical stranglehold offers a new perspective on the history of forensics.

Our choice of historical case study to accomplish this might appear an unlikely one, as Gross and his *Handbook* have gone largely unnoticed by historians. By contrast, practitioner accounts of the rise of forensic science in general, and of scientific approaches to criminal investigation and policing in particular, routinely pay tribute to its status as a formative text. Its publication was a ‘watershed event’ according to a recent assessment, ‘the first comprehensive textbook to systematically cover the integrated philosophy and practice of scientific criminal investigation, forensic analysis, and crime reconstruction. Its philosophies have not been diminished by the passage of time and should be required study for any student of these subjects.’^6^

Celebratory assessments in the practitioner literature come as no surprise, for they respond to the elements of the Grossian regime that have become routinised in contemporary crime scene investigation (hereinafter CSI): the identification and preservation of trace evidence, the avoidance of scene contamination, chains of custody, and the interface between the field and the laboratory, amongst many others. In our analysis we will attend to facets of this recognisably ‘modern’ Gross. But we will also give detailed consideration to a key feature of his handbook that does not so easily map onto present-day expectations: the multilayered, synoptic role assigned to the investigative enterprise’s central character: its eponymous *untersuchungsrichter* (Examining Magistrate, or, following the English translations, Investigating Officer). To be sure, this can in part be explained by reference to organisational differences of forensic culture: Gross’ Investigating Officer (hereinafter IO), a judicial official charged with overseeing, co-ordinating, and personally conducting investigations into criminal cases, does not have an equivalent in the Anglo-American world of CSI. This difference in function for our present purposes is of marginal interest. However, it does engender the feature of Gross’ text that is a core concern: its provision of a detailed, and strikingly self-reflexive, account of the physical, physiological and psychological considerations that underpin—and equally threaten to undermine—even the most ostensibly simple act of crime scene perception.

Attention to the way in which Gross constitutes his IO as a reliable observer and manager of hidden crime scene traces is crucial to our dual objective of historicising the crime scene and thereby enabling criminalistics to emerge from the under shadow of criminology. We should here acknowledge that this involves a degree of selective attention. Like Bertillion and Galton, historically and historiographically, Gross occupies a hybrid position between criminology and criminalistics. His allegiances to the former are formidable: as professor of criminal law at the Universities of Czernowitz (1897-1902), Prague (1902-1905) and Graz (1905-1915), he was deeply engaged in contemporary debates on criminality as an anthropological and psychological phenomenon.^7^ In 1898 he published his influential *Criminal Psychology*, which despite its systematic critique of Lombroso’s empirical failings and its championing of psychologically-driven research into perception and memory as an alternative to the dominance of criminal anthropology, still worked within a broadly degenerationist framework.^8^ In the same year he founded the journal *Archiv für Kriminalanthropologie und Kriminalistik* (*Archives for Criminal Anthropology and Criminalistics*), which over the course of his nearly twenty years as editor in chief developed an international reputation as an outlet for advanced research in the field.

As the title of his journal suggests, however, there is different side to Gross: not the author of *Criminal Psychology* but of *Criminal Investigation*, published five years earlier.^9^ The genealogy of this book is linked to his work as a professional crime fighter that preceded his academic career. Having completed his law studies in his home town of Graz in 1870, Gross was appointed as an IO for Upper Styria. It was in this capacity that he accrued two decades worth of practical experience of engaging in the investigation of specific crimes and the pursuit of their perpetrators. In a series of articles the historian Peter Becker has provided a valuable contextualising framework for this version of Gross, one that helps us to locate our analysis of Grossian CSI within a set of disciplinary and practical trends in continental criminology.^10^ Becker places Gross as part of a generation of German-speaking police and law reformers in the second half of the nineteenth century whose main aim was to set the modern investigation and prosecution of crime on a standardised, objective footing. They attempted this in large part by modelling their practices on the contemporary physical and natural sciences, whose characteristics Becker derives in large measure from Daston and Galison’s classic work on the interlaced cognitive, technical and emotional disciplines associated with an emergent regime of ‘mechanical objectivity’.^11^

Our understanding of Gross largely supports Becker’s analysis, in that we see Gross’ attempt to present a system for criminal investigation as a composite of individual affect, gesture and perception, and networked bureaucratic routine. We differ, however, in our selection of the site at which to observe and analyse this set of practices. Becker’s principal interest lies in the way that this reformed regime engages with its human quarry—the bodies, mind and ‘lifeworlds’ of criminals themselves. Thus, for instance, he argues that the standardisation of methods of criminalistic observation, ranging from physiognomic description to the photographic capture of criminal types, lent the legitimising aura of the physical and natural sciences to the control and surveillance of criminal deviance.^12^

By contrast, in what follows we use Gross to articulate a criminalistic-centred history of forensics. We do this fully aware of his criminological side, which is in plain view in *Criminal Investigation*: chapters devoted to criminal slang and superstition, the habits of ‘wandering tribes’, and other staples of criminal anthropology and psychology occupy roughly one-fifth of the text. In addition, even in the sections devoted to the IO’s crime scene activities, the discussion of traces (much like Bertillon’s treatment of ears) can take on a dual significance. Excrement left at crime scenes, to take a particularly striking example, has at the same time an anthropological dimension (a superstitious practice of certain criminal ‘tribes’) and one relevant to crime scene analysis: excrement can be examined for its constitutive physical traces (seeds, parasites, e.g.) that may link it to its human source.^13^ It is to this latter version of crime scene trace—and to the conditions for its effective investigation—that our discussion now turns.

## 2 Preparing the investigator: Gross and the psycho-physiology of perception

The first line of Gross’ *Handbook* makes its author’s objective crystal clear: ‘The aim of this book is to be as practical as possible.’ Neither a law book nor a traditional work of medical jurisprudence, Gross continues, it is instead a manual of instruction ‘for all those engaged in investigating crime’.^14^ Yet its sheer scale—which in the first English edition ran to over 900 pages culminating in a ‘selected list of authorities’ comprised of more than 1000 entries—signals that this was no straightforward set of common-sense guidelines. For Gross, ‘practical’ action, effectively executed, was anything but straightforward. An individual’s most basic perceptual and cognitive actions were deeply mediated, and these levels of mediation, and their potential to mislead, needed to be systematically analysed in order for effective criminal investigation to take place.

Still in the introduction, Gross begins to unpack this world of complex mediation. He starts with what at first glance looks like a secure and in his day an increasingly commonplace opposition: between the testimony of human witnesses, and the testimony of physical things. Human testimony, even that of the most well-intentioned witness, is in his view ‘much over-rated’, because it is subject to a myriad of distorting influences—the physical and emotional status of the witness or the physical conditions that frame an event, for example. He then, and again at first glance entirely conventionally, turns to the comparative value of material (in his words ‘realistic’) proof:

The trace of a crime discovered and turned to good account, a correct sketch be it ever so simple, a microscopic slide, a deciphered correspondence, a photograph of a person or object, a tattooing, a restored piece of burnt paper, a careful survey, a thousand more material things are all examples of incorruptible, disinterested, and enduring testimony from which mistaken, inaccurate, and biassed perceptions, as well as evil intention, perjury, and unlawful co-operation, are excluded.^15^

So far, so simple. But Gross’ more challenging notion of the ‘practical’—into which the reader will soon be drawn—is signalled by the immediately preceding sentence: if psychology teaches us to be attentive to the sources of distortion in human testimony, he wrote, ‘so the other parts of the subject show us the value of facts, where they can be obtained, how they can be held fast and appraised—these things are just as important as to show what can be done with the facts when obtained.’^16^ In other words, the identification, retention and evaluation of *physical* evidence are acts of perception and cognition that equally require self-scrutiny to be effective. It is important to note at the outset, then, that Gross’ text does not operate on a pre-established privileging of things over humans (though it does operationalise this as a legitimate hierarchy once properly justified). Instead, it is treated as the outcome of a way of seeing and acting that creates the conditions for things being able to speak for themselves.

In the context of criminal investigation, the lynchpin of ‘incorruptible, disinterested, and enduring testimony’ is the IO, and thus it comes as no surprise that the first part of the *Handbook* is entirely devoted to sketching out this crucial figure. His heroic general attributes (possession of ‘the vigour of youth, energy ever on the alert, robust health … liveliness and vigilance’) are supplemented by a formidable intellectual capacity, an ability ‘to solve problems relating to every conceivable branch of human knowledge’, and mastery of a range of esoteric topics (e.g. knowing why boilers explode, how to read account books, understanding slang and decoding ciphers).^17^ In outlining these essential characteristics Gross emphasises their broad and practically engaged nature—anything and everything may be of use in his line of work, so from the moment of taking up the role the IO becomes a student of the world and everything in it. Importantly, this omnivorous attitude is an on-going matter of disposition enacted prior to any specific investigative engagement: ‘He who seeks to learn only when some notable crime turns up’, Gross warns, ‘will have great difficulty in learning anything at all. His knowledge should be acquired beforehand by constant application in his ordinary life. Every day, nay every moment’, he continues,

he must be picking up something in touch with his work. Thus the zealous Investigating Officer will note on his walks the footprints found on the dust of the highway; he will observe the tracks of animals, of the wheels of carriages, the marks of pressure on the grass where someone has sat or lain down, or perhaps deposited a burden. He will examine little pieces of paper that have been thrown away, marks or injuries on trees, displaced stones, broken glass or pottery, doors and windows open or shut in an unusual manner. Everything will afford an opportunity for drawing conclusions and explaining what must have previously taken place.^18^

By engaging in this practical observation and the causal inferences that ordinary phenomena suggest to the attentive observer, the IO trains for the moment when these background skills are called upon in the face of the extraordinary—the scene of a crime.

Throughout this discussion the theme of ‘preparation’ recurs. Cognitive preparation is accompanied by a physical and spatial one, through which the IO embeds himself within a known context from which information and resources, once required, can be readily called upon. Thus, in his everyday ramblings he will have as ever-present companions instruments (e.g. an ordnance survey, a watch, a compass) enabling him to establish objective coordinates such as relative distance and travel time between fixed points. Indeed, Gross includes a named subsection on ‘orientation’, in which he instructs the IO on methods for assembling in advance his ‘base of operations’—including a network of subordinates and auxiliaries (from police officers and grave-diggers to local medical men and university-based experts) on whom he could draw with confidence in the event of an occurrence requiring investigation.

There are obvious practical dimensions to this preparatory attitude, but underneath it lurks a constant theme: preparation aids the IO to be an effective observer by helping shield him from the vicissitudes of perceptual and cognitive error. In this way, Gross presents the translation of the place in which a crime occurred into a ‘crime scene’ as dependent on, and interactive with, a *prior* set of labours undertaken by his IO. The crime scene and IO are co-extensive products, called into being by the thoroughness of Gross’ practical advice, and dependent not merely on material requirements but their human correlatives as well.

Attention to this interplay between observer and observed, investigator and material trace, results in a collapsing in his text of the ostensibly straightforward opposition mentioned above between the testimony of things and of human beings, an opposition that figures both in the writings of Gross’ contemporaries and in modern histories of *fin-de-siècle* forensics.^19^ The point of departure for both is the significance attributed to research into the psycho-physiology of perception from the mid nineteenth century, initially in the German laboratories of investigators like Hermann von Helmholtz and Carl Ludwig and then by a second generation of researchers, most notably Hugo Munsterberg in the U.S. The core insight of this work—that the dynamics of memory, cognition, experience and the like may lead even the most honest human witness to provide false testimony—had two linked results. First, it led to the recognition that everyone involved in the investigation and prosecution of crime needed to be aware of the causes and implications of witness perceptual fallibility, and to adjust their strategies for eliciting and interpreting witness statements accordingly. Second, commentators elevated material over human testimony, seeking out ‘mute witnesses’ wherever possible to short-circuit the evident dangers of relying on the human alternative.^20^

But in developing his manual Gross added a complication to this simple reversal in the evidentiary hierarchy: yes, the IO needed to be instructed in the vicissitudes of human testimony; and yes, physical evidence was invaluable and often more reliable than its human counterpart. Yet making matter testify accurately was itself a complex task fraught with possibilities for error. This is because research into the contingencies of cognition and perception applied as much to the IO as to any other testifier. For the crime scene to speak in a secure language of material fact, in other words, its interlocutor needed to be prepared in advance to receive and appreciate its meaning. It is in this sense that the Grossian vision of CSI entails interdependent human and material work. The protocols of CSI that at first glance appear to merely secure the material objects at the scene (the careful delineation of a space of investigation, the physical restraint in approaching and handling objects in that space, the meticulous graphic recording of those objects in their spatial and temporal dimensions) at the same time serve to secure the IO as a reliable harvester of trace evidence.^21^

This background work prepares the IO to confront, and conquer, what Gross identifies as ‘the most deadly enemy of all inquiries’—preconceived theories. These, Gross advises his reader, will be shown throughout the volume to be numerous in origin, astonishingly easy to take root, and exceptionally difficult to extirpate.^22^ As a rule, preconceived theories result from examining an issue ‘from a false point of view’. This can happen, Gross explains, for physical reasons—objects can appear different from what they really are, for example, either by virtue of a faulty sensory apparatus (e.g. physical pressure on the eyeball) or by the conditions in which the perception took place. To illustrate this latter point Gross invokes the effects created by observations in a humid atmosphere. Through water charged air, mountains appear closer than they really are, an effect for which he rejects the term ‘illusion’ because it conforms to known physical laws. The same applies to the classic example of the ostensibly bent stick halfway submerged in a glass of water. Here again, Gross insists, the fact that the stick appears bent is not itself an illusion—it is only when someone, ignorant of the principles of optics, thinks that the stick is in reality bent, that accurate observation is threatened.^23^

But there were more subtle, and dangerous, ‘moral’ sources of error. By way of illustration Gross offers a hypothetical case in which an IO is informed of a case of arson in a distant locality:

immediately in spite of one’s self the scene is imagined; for example, one pictures the house, which one has never seen, as being on the left-hand side of the road. As the information is received at headquarters the idea formed about the scene becomes precise and fixed. In imagination the whole scene and its secondary details are presented, but everything is always placed on the left of the road; this idea ends by taking such a hold on the mind that one is convinced that the house is on the left, and all questions are asked as if one had seen the house in that position. But suppose the house to be really on the right of the road and that by chance the error is never rectified; suppose further that the situation of the house has some importance for the bringing out of the facts or in forming a theory of the crime, then this false idea may, in spite of its apparent insignificance, considerably confuse the investigation.^24^

At its root, Gross continues, this error of the imagination follows from a ‘psychical imperfection’ inherent to humans, who at a general level are interpreters rather than recorders, interpreters who bring their own predispositions to bear on any given act of observation. They tend to complete stories, to fill in gaps (perceptual and/or cognitive) with a bank of pre-stored data that supplement what is registered via direct experience.

There are several consequences to this, none more important than the ordinary observer’s penchant for noticing, and prioritising, that which appears striking and vivid:

it is only in conformity with human nature to stop the more willingly at what is more interesting than at what belongs to everyday life. We like to discover romantic features where they do not exist and we even prefer the recital of monstrosities and horrors to that of common every-day facts. This is implanted in the nature of every one, and though in some to a greater, in some to a lesser extent, still there it is. A hundred proofs, exemplified by what we read most, by what we listen to most willingly, by what sort of news spreads the fastest, show that the majority of men have received at birth a tendency to exaggeration.^25^

In itself, Gross observes, this is no great evil—indeed, it is the well-spring of the creative imagination. But the IO ought, by definition, to be a class apart: ‘in the profession of the criminal expert everything bearing the least trace of exaggeration must be removed in the most energetic and conscientious manner; otherwise, the Investigating Officer will become an expert unworthy of his service and even dangerous to humanity.’^26^ Here the IO’s background habituation to noticing the ordinary comes into its own—he knows that what appears the most striking is not necessarily the most significant. But adhering to this attitude requires constant work: ‘The only remedy is to watch oneself most carefully, always work with reflection, and prune out everything having the least suspicion of exaggeration.’^27^ In other words, the IO’s acts of perception are subject to the same sources of error as those of any other witness. Information given to or unearthed by him is registered through his own perceptual apparatus, and is thus subject to the very distortions that threaten any product of the act of witnessing. As a necessary consequence, the ostensible objectivity of material traces is an outcome, rather than a prior condition, of the procedures Gross is delineating.

This outcome ultimately depends on the IO’s ‘submission to severe discipline’, one that comes into its own at the crime scene.^28^ It is to this novel space that we now turn. It is important to emphasise that in doing so our aim is to both describe the making of the crime scene and to show how its constitutive elements (its gestures, sensibilities, routines of investigation, recording and preserving) reinforce Gross’ characterisation of the IO that we have just outlined. The instructions he provides on the proper way of effecting CSI thus serve a double function: on the one hand they can be considered generic (at times banal) homilies on the importance of investigative care. But the constant self-monitoring is also essential to the physical and cognitive practice of CSI. In other words, his engagement with the problems of perception that we’ve sketched above are a logical and operational prerequisite for the crime scene’s epistemic status as a field of latent, objective material traces that can be utilised as such for the purposes of investigation. The crime scene as a space of hidden but objectively apprehendable traces, therefore, is not merely the site for the deployment of a highly structured way of seeing, but is in fact *produced* by it.

## 3 CSI: Suspending space and time

Gross’ preliminary strictures on the dynamics of perception, the mediations of the imagination and the need for submission to forms of physical and cognitive discipline are all put into action in the subsequent sections of the *Handbook*. The first of these focuses on witness testimony, in which he provides a comprehensive outline of what the IO needs to consider when gathering and evaluating evidence based on human perception other than his own. But it is his next section, ‘Inspection of Localities’, that most interests us, for it is here that Gross at once calls into being a new space—the crime scene—and aligns his earlier observations to it as a necessary precondition for its successful management.

There are several interlocking elements of Grossian CSI that we will outline generally, and then subject to further critical scrutiny. First, the crime scene is a space of record: the IO’s report, Gross stipulates in the opening line of this discussion, ‘is a real touchstone’.^29^ Second, despite its often alarming elements (e.g. blood, corpses), the crime scene is a space of emotional and physical equilibrium. Third, it is a space of mental and gestural restraint: mentally, it has no place for preconceptions as to what transpired within it, which of its material traces are significant, or what they might signify; gesturally, it demands suspension of any impulse to engage (and thus potentially disturb) that which appears to be of interest and value. As he develops these three virtues of CSI, they interlace to simultaneously construct a space of investigation and align its investigator to it, and in so doing call into play the preparations and self-understanding that has gone into the making of a Grossian IO.

The opening lines of the section ‘What to do at the Scene of Offence’ capture the essential relationship between investigative space and investigator affect: ‘On arrival at the scene of the crime certain things must be attended to which are common to all cases, be they of simple theft, robbery, murder, arson, or misdemeanour. The first duty is to preserve an absolute calm. With it everything is won, without it everything is compromised.’ This calm serves several functions. First, it enables the IO to efficiently activate his network of auxiliaries, thereby preserving a key feature of his prior orienting labour:

An Investigating Officer who fusses about, sets to work aimlessly, starts a plan only to drop it, asks everybody useless questions, and gives orders only to cancel them, makes a most painful impression on those engaged with him in the inquiry and destroys any confidence they may have had in his successful management. . . . But if the Investigating Officer shows perfect confidence with no trace of excitement, and acts as with a sure prevision of the results, everyone willingly submits to his orders and each does his very best and the result of the enquiry is assured.^30^

Calm also affords the IO with an initial opportunity for self-scrutiny. Echoing his earlier discussion of the tendency to project and fix an image of phenomena in advance, Gross warns that the very act of being called to the scene itself represents a possible layer of mediation:

As soon as the Investigating Officer is informed about a case it absorbs all his thoughts … [H]e immediately makes a mental picture of the case itself and all connected with it, in a definite form, with precise outlines; when travelling to the spot he bases upon this idea his conjectures as to how the offence has been committed, and builds, upon his mental picture of the spot, the plan of inquiry to be pursued. The idea may take root in his mind to such an extent that he cannot rid himself of it either in part or in whole even when the scene is actually displayed before his eyes…

Calm at the start of his physical engagement with the scene enables a realignment of these preconceptions—or, in Gross’ words, to ‘find his bearings’.^31^

Having thus oriented himself, the IO is ready to shift from background to foreground work, but again this work is informed by his prior submission to Grossian rigour. His first task is to secure the crime scene by fixing it as a spatio-temporal entity: ‘The exclusion of everything happening after the moment when the crime was committed’, he explained, ‘is a very special task for the Investigating Officer’. This had several components that have since become CSI staples: marking off a perimeter zone to prevent unauthorised access, and shielding ‘vestiges of the crime’ within it from contact with forces that might degrade or contaminate them, for example.^32^ In doing this the IO enacts his suspension of ordinary standards of distinguishing between, and reacting to, what seems noteworthy and what trivial:

everything may be of importance and nothing too small or insignificant to have a decisive bearing upon the case. The situation of an object an inch or two to left or right, to front or back, a little dust, a splash of dirt easy to efface, may all turn out to be of the first importance.^33^

This realisation enforces a set of gestural disciplines which secures the crime scene as a space of latent meaning, meaning inherent, moreover, not merely in the things lying within it but, crucially, in their relationship to one another. The crime scene thus requires protection against the ‘natural impulse’ of those who engage it, that is,

to immediately touch any object of apparent significance, as e.g., an object left on the scene of the crime by the criminal. It is laid hold of and moved about, and only afterwards is it recognised that the object in itself signifies very little but that everything depends on its position–which can no longer be fixed.^34^

Gross illustrates this with an appropriately striking example, one that underscores his concern to contain the destabilising effects of the emotive dynamics of perception and cognition. When confronted with a murder victim, he writes, the ‘involuntary impulse’ is to seize the hands and search them for signs of a struggle (hair, torn clothing, e.g.). But by thus indulging the desire to assemble narrative out of obviously compelling elements, the undisciplined IO sacrifices a world of more subtle clues and thus projects himself into the crime scene not as its protector but as a prime source of degradation and contamination.^35^

By contrast, his Grossian counterpart follows his mentor’s ‘golden and inviolable rule’: ‘*Never alter the position of, pick up, or even touch any object before it has been minutely described in the report*.’^36^ This dictum draws attention to the central place of recording in CSI. First, it showcases the critical importance of the crime scene report as a means of capturing (however fleetingly) a moment in time and space, a moment that, despite the IO’s best efforts, can only ever be temporarily achieved.

**Fig. 1:**
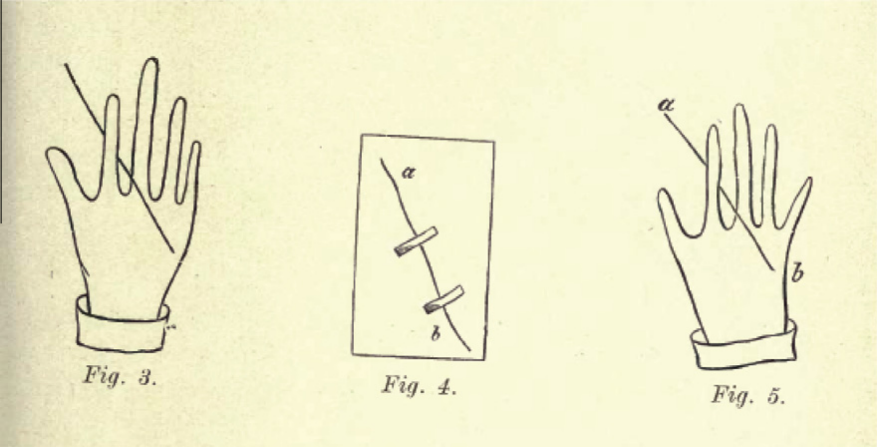
(Gross 1906), p. 196.

Gross lavishes elaborate (and tedious) detail on how to record in order to preserve, and suggests a series of aids to this end. In the classic case of hair found clapsed in a murder victim’s lifeless hand, he provides an ‘intentionally primitive’ series of drawings for registering spatial and relational details—e.g. the direction of the hair root—that went beyond the mere fact of presence.^37^ (see Fig 1) Elsewhere he furnishes more sophisticated instruction, insisting on conformity to a ‘technical formula’ and the utilisation of objective reference points secured instrumentally (compass points, spirit level readings, plumb-line angles). Thus, for example, in sketching a room in which a murder had been committed, Gross instructs the IO as follows:

the door should be taken as a starting point and the same direction followed as the hands of a clock, i.e., standing in the entrance and facing into the room, start from the left hand and go round the room towards the right hand; in this way one will be certain that nothing has been forgotten. First describe the size, shape, height, and other peculiarities of the space in question. Then go from the entrance towards the nearest left corner, then the left-hand wall, then the wall facing the entrance, then the right-hand wall, then the remainder of the wall to the right of the doorway, and finally the objects in the middle of the room. In the course of this description the windows and the doors will be noticed. Next describe any alterations in the state of the moveables in the room consequent upon the crime in question, damage done by blows, bloodstains, changes in the situation or position of objects, damage to windows, doors, etc; and finally a minute description of the subject-matter of the crime (e.g., a broken safe, a dead body, etc.) with all the particulars necessary to a detailed description.^38^

**Fig. 2:**
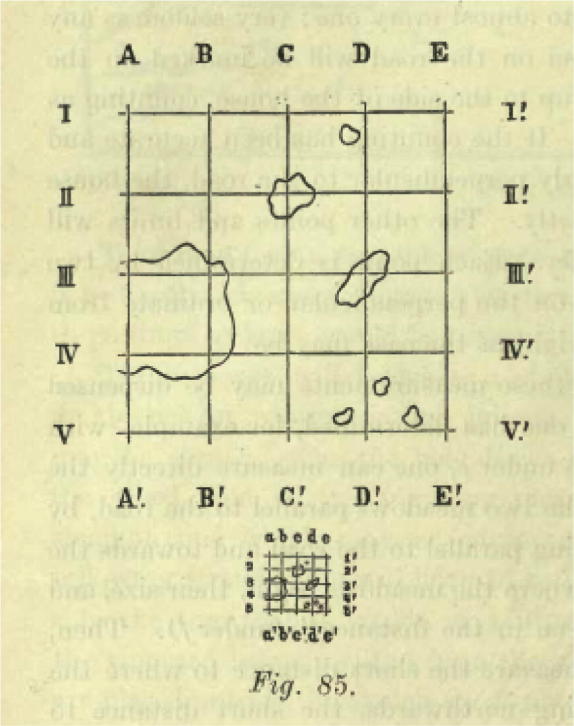
(Gross, 1906), p. 468.

A later chapter on drawing (as one of the IO’s ‘special crafts’), provides still further detailed instruction on how to secure objective ‘orientation’ in crime scene sketches, advising the re-plotting of the physical space of the crime scene onto squared paper, and the reproduction in the paper’s squared units of ‘everything that may be seen in nature in the large squares’. Gross illustrates this (see Fig 2) with the following explanation:

Suppose that a certain number of drops of blood which have dried upon a plank have to be depicted. The large sketch represents the actual subject-matter and the small one the finished drawing. First examine the portion of the plank to be drawn and its dimensions. This is then delimited by means of a set-square, setting down the lines A A’ & I I’ at right angles. These are then divided into a certain number of equal parts, the more there are of them the more accurate the result will be; parallels are then drawn so as to obtain a certain number of squares of equal size.^39^

These instructions at once reinforce and encode in practice Gross’ general strictures against rushing to examine what initially strikes the IO as the most interesting, or significant, element of the scene. Translating the natural space of the crime scene onto a paper grid effects a regime of total recording: it is only at the end of an exhaustive description of the scene as a whole that crime’s ‘subject-matter’ is considered. The investigator who systematically records rather than touches thus secures the crime scene for future analysis at a distance in time and space. But in addition he confirms his physical and cognitive self restraint, his capacity to defer engagement with the world of immediate appearance in order to preserve one of as yet unseen, fragile traces and inter-relationships. It is this set of practices that instantiates a modern regime of CSI, with its imperatives of preservation of matter in time and space, of guarding against physical and conceptual contamination, of guaranteeing the future use value of harvested material through routine gestures. Crime scene recording, then, is at once the outcome of investigative discipline and its guarantor.^40^

Moreover, and in keeping with our interest in the embodied gaze that anchors this web of practices, we can take this set of fundamental gestures as providing a further means of ‘orienting’ the IO. The very act of minute recording ‘with scrupulous exactitude the description of how everything is found on the spot’ produces an observational attitude prepared to detect minor ‘errors’ or discontinuities in a scene of ostensibly conventional appearances:

So long as one only looks on the scene, it is impossible, whatever be the care, time, and attention bestowed, to detect all the details, and especially to note various incongruities: but these strike us at once when we set ourselves to describe the picture on paper as exactly and clearly as possible.^41^

To develop this point Gross turns to a technique he attributes to painters, in which they seek out flaws in their compositions by looking at them in a mirror, which induces visual estrangement from a scene that they have grown accustomed to viewing from a single perspective. By recourse to such an inverted image of their work, ‘defects in a drawing are most easily and surely detected’.^42^

In CSI, scrupulous recording serves an analogous function: ‘the exact description of the surroundings is so to speak the mirror in which all “defects of the situation” are reflected.’^43^ Defects, again, derive from perceptual distortion caused by preconception—from being habituated to one way of seeing, or indeed by imposing one’s own narrative expectations or assumptions. Such impositions, Gross explains, will produce contradictions (‘defects of the situation’) which remain invisible to the perceiver unless some mechanism of estrangement intervenes. Indeed, this fixed (and ultimately flawed) conventionalised way of seeing abets the perceiver’s distorting desire for an explanatory account of what is before him. It takes effort to disrupt this tendency, to dislodge the human tendency to see within a conventionalised frame of meaning. It is the mirror that makes these discontinuities—between what a viewer sees through eyes that seek simple explanation of what is seen and the more complex realities of what is actually there—evident to those who submit to its discipline: ‘the “defects of the situation”’, Gross explains, ‘*are just those contradictions, those improbabilities, which occur when one desires to represent the situation as something quite different from what it really is*’.^44^

Gross makes a similar point when earlier in his text he invokes the practices of the copy-editor whose ability to detect textual errors depends on his suspension of conventionalised reading habits. When we read, he observes, we do not notice every letter, but take in the whole word as a general ‘shape’ that conforms to a prior bank of comparative forms. As a consequence, we constantly fail to notice minor printer’s errors, especially if the word is long and the mistake does not markedly modify its appearance.

Something analogous occurs in all perceptions and more frequently than we ordinarily suppose: what enables us to seize more easily the aspect of a whole is that we seek and store up in our memory certain characteristic features from which we can immediately spot the object.^45^

In his reading of the crime scene, the IO similarly requires an eye that resists the embrace of conventionalised meaning and instead seeks out anomalies that identify the particularised ruptures to which trace evidence alludes.

Gross’ routine CSI practices thus articulate with his foundational observations about the conventionalised nature of perception, the tendency for observers, even with the ‘very best intentions’, to view a scene through a prior bank of conceptions. Minute recording and its allied physical and conceptual disciplines confirm the IO’s position as an actor apart: they simultaneously preserve evidence in time and space, stabilise the IO’s emotional register, and protect against the impulses and indeed the vision stemming from an all too human tendency to engage at the level of story.

## 4 Concluding thoughts: Historicising CSI

In this article we have examined the late nineteenth-century origins of crime scene investigation as developed by Hans Gross. In demonstrating the debt that our present-day criminalistics-centred forensic culture owes to Gross and to the historical moment in which he was embedded, we also wanted to raise a related historiographical point about the value of repositioning the history of forensic theory and practice outside of the parameters of *fin-de-siècle* criminology. Our analysis of his contribution involved an appreciation of its engagement with late nineteenth-century questions about perception and cognition as a complex psycho-physiological process. Gross may well be an important forbear of the gestures and sensibilities of modern CSI, but he is also much more than that. His work provides a vantage point from which to consider the historical contingencies of CSI and to understand how the desire to overcome the ambiguities and interpretative flexibility inherent in human perception was, at least for one its progenitors, a motivating force behind the pursuit of a trace-centred forensics at the turn of the twentieth century.

By way of a conclusion, we want to reinforce our argument by (briefly) considering Gross’ discussion of the uses of photography at the crime scene. Today, crime scene photography, with its ability to create a permanent record of often evanescent objects and their relation to one another at a fateful moment in time and space, is one of CSI’s defining tools.^46^ In certain respects, Gross’ views on the camera’s crime scene role can be easily transposed onto our own modern forensic expectations. He gives broad backing to the camera’s value at the crime scene as a means of supplementing the IO’s quest for total recording and preservation, citing with approval the forensic chemist Paul Jerserich’s assertion that photography ‘is entirely objective and always impartial; it is capable of fixing certain details which may perhaps be of subsequent importance and of which no one has dreamed at the time of the inspection of localities.’^47^

However, and taking his lead from psychological and philosophical discussions about the nature of perception, Gross refused a simple equation of photograph and truthful reproduction. He signals his interest in complicating this view in a footnote to the Jerserich passage, which acknowledged the existence of a contemporary debate about the epistemic status of photographic reproduction: ‘Concerning the objectivity and the apparent untruthfulness of photography’, Gross remarked, ‘too much cannot be said. We must try and find out why photographs frequently create a wholly wrong impression’.^48^

What was also significant about Gross’ set of observations on photography was its dovetailing with his overall vision of CSI. First, like the discipline of meticulous description, which enables the IO to resist the seductions of conventionalised vision, crime scene photography creates a perspectival space that enables reflection and correction. Invoking again the example of the artist seeking to escape habituated vision by recourse to a mirror image, Gross proceeds to make a direct link:

In photography exactly the same may be said; an object has been observed with great minuteness and application; a whole series of observations have been made regarding it; nothing striking has been noticed about it because one has become accustomed to its appearance; but if it be photographed, the new colour, the new situation, and the new aspect enable us to see it from another point of view and reveal fresh details which have not yet been discovered.^49^

In this sense the apparent ‘distortion’ inherent in photography becomes a resource for CSI, the photograph furnishing the IO with an enabling ‘paradox’: it ‘shows us more than the eye, even when it shows us no more than the eye can see.’^50^

The photographic image as a technically mediated product holds out further possibilities for extending the vision of the IO. In processing colour, for example, the photographic process renders reds and browns darker and clearer, and even makes them visible when a human eye cannot see them at all. This had implications for certain types of forensic investigations—in examining a body for signs of bruising, for example, photography could reveal ‘latent’ brown and red marks of potential evidentiary value: ‘Every pressure exercised on the skin of a man’, Gross explained, ‘results in the breaking or at least in the inflaming of the small veins, and each time redness is produced. If the pressure has been very feeble the redness will exist objectively but will not be discernible by the eye.’^51^

Turning to the apparent ‘inadequacies’ of photographs, Gross identifies and illustrates a fundamental difference between human and mechanical vision. When a person places one hand in front of the other so that it is closer to an observer sitting opposite, for example, that observer will perceive the hands to be of equal size. However, if the hands are photographed from the observer’s perspective, in the resulting photograph the hand nearest the camera will appear larger than the other. This effect, he explained, captures the physical reality of the situation as governed by optical laws. However, in everyday practice our sense of visual realism is governed by different laws, ones drawn from everyday experience, that influence observation and lead us to draw the opposite conclusion:

We know the two hands are really of equal size and this knowledge has so powerful an action that we see them of equal size although, being in perspective, they ought to appear unequal. On looking at the photograph of the hands, however, the principle of experience no longer acts with the same force; on the contrary, we remember that what we are looking at is a picture and attribute the fault to it and say it is an inaccurate reproduction, for we see upon the picture an exact reproduction from the point of view of perspective and we notice an enormous difference in size, but it does not seem to agree with the reality.^52^

In photography, as in all matters of crime scene investigation, seeing effectively requires disciplined self-awareness that cannot be achieved through mechanical means alone.

More broadly, as we have argued throughout, understanding Gross’ remarkable attentiveness to the interrelated physical, perceptual, cognitive and emotional dynamics that govern the IO’s labours is critical to revealing how the crime scene emerged at the turn of the twentieth century as its own epistemological space. Engaging the Grossian project on its own terms enables an understanding of crime scene investigation as a historically and culturally variable set of practices. In so doing—by historicising the processes and strategies through which early advocates like Gross sought to establish the authority, legitimacy, and necessity of a trace-driven mode of forensic investigation, we can also avoid making determinist assumptions about its inevitability. Exploring what its proponents wanted to achieve with CSI, and what they believed was required to put their vision into operation, opens up a fresh pathway for pursuing forensics past, and present.

## Footnotes

1 Davie (2005), Gibson (2002), Nye (1984), Pick (1989), Wetzell (2000).

2 This is a point made by Claire Valier and, more recently, Daniel Mark Vyleta, who criticise the historiographical framing of criminology as a scholarly discipline for its over-emphasis on the mind, body, and milieu (social, environmental) of criminals as a way of identifying them, rather than the development of scientific methods of investigating individual instances of crime. Valier (1998), Vyleta (2006).

3 Sekula (1986), p. 19. ‘“Criminology”, in his succinct phrasing, ‘hunted “the” criminal body. Criminalistics hunted “this” or “that” criminal body.’ (p. 18).

4 Cole (2001), p. 100.

5 *Ibid*.

6 Chisum and Turvey (2011), pp. 30, 32.

7 For biographical details, see Bachhiesl (2010), Becker (2004), Grassberger (1956).

8 Gross, 1911 (1898 orig.). For a critical assessment of Gross’ historiographic treatment as a Lombrosian acolyte, see Vyleta (2006).

9 As Becker points out, however, the German term ‘*Kriminalistik’* refers to the broad field of professions engaged in matters of criminal law, and thus has a more generic usage than the one we are adopting for its English counterpart. Becker (2001), p. 199.

10 Becker (2001, 2004, 2005, 2006).

11 Daston and Galison (1992, 2007).

12 Though Becker does suggest that the objectifying trajectory he is examining with respect to criminals had an analogue in the materiality of criminal investigation, this is not his principal interest, and thus he does not attempt to engage it systematically. The closest convergence between Becker and ourselves comes in his article juxtaposing the traditional criminological and innovative criminalistic elements of Gross’ *Handbook*. Becker’s observations on this latter version of Gross resonate with elements of our analysis, though by displacing the criminological Gross to a greater degree we are able to provide a fuller analysis of Grossian CSI. Becker (2004).

13 Gross (1906, 1893 orig.), pp. 193–194, 383–384. This hybrid quality of Gross’ text has been noted by Christian Bachhiesl (2010) with respect to crime scene blood traces (as both an object of bio-chemical and anthropological analysis), and by Becker (2004), with respect to footprints (as measureable physical trace and characterological indicator), for example.

14 Gross (1906), p. xxi.

15 *Ibid*., p. xxvi.

16 *Ibid*.

17 *Ibid*., p. 1.

18 *Ibid*., pp. 2, 3. This feature of Gross’ text is also discussed in Becker (2004).

19 For a nuanced historical account of *fin-de-siècle* legal and scientific discussions on the relative value of human and material testimony, see Golan (2006), chapter 6.

20 Gross’ engagement with the psychology of perception provides an opportunity to note a suggestive link between his conceptualisation of mental and physical investigation. In *Criminal Psychology* he makes the following observation: ‘“Every state of consciousness has its physical correlate,” says Helmholtz, and this proposition contains the all in all of our problem. Every mental event must have its corresponding physical event in some form, and is therefore capable of being sensed, or known to be indicated by some trace.’ Gross (1911), p. 41. Here Gross is urging the investigation of unconscious and involuntary physical behaviour—e.g. reflex responses, nervous laughter—to unmask deceitful criminals. But in doing so he also establishes a striking parallel between the *mind* (as a repository of retrievable, physical evidentiary traces created by its engagement with the external world), and the *crime scene* (as a space for the retrieval of another set of significant physical traces).

21 Here we diverge from Becker’s analysis, which notes the engagement of Gross and his ilk with the contemporary literature on the psychology and physiology of perception, but which then dismisses it as a half-hearted, largely rhetorical exercise. The reason he gives for this assessment is the essentially criminological nature of their interests—‘the classification and reconstruction of human behaviour’—which would not bear the scrutiny of a scientifically rigorous account of the preconditions for objective perception. In our analysis, the reverse is the case: Gross’ engagement is direct, detailed, and integral to his overall project. Becker (2001), esp. pp. 227–228.

22 Gross (1906), pp. 5, 14-5.

23 *Ibid*., p. 64.

24 *Ibid*., p. 16.

25 *Ibid*.

26 *Ibid*.

27 *Ibid*., p. 17.

28 *Ibid*.

29 *Ibid*., p. 123.

30 *Ibid*., p. 126.

31 *Ibid*., pp. 126,127.

32 *Ibid*., p. 128.

33 *Ibid*., p. 130.

34 *Ibid*.

35 *Ibid*., p. 130. The eschewal of conventional expectation equally informs the IO’s approach to the search for evidentiary traces. ‘There is little probability of finding anything of importance if the attention be confined to safes, almirahs, beds, boxes, stoves, or chimneys’, Gross warns. ‘Absolutely everything must be examined, for there is no place where important objects cannot be hidden.’ *Ibid*., p. 163. Similarly, the IO’s report needed to suspend common narrative standards of interest by including not merely positive evidentiary finds but also their absence: ‘The notification of even purely *negative* facts should not be neglected, for on the one hand they may lead to positive inferences and on the other reassure the reader and show him that they were not forgotten altogether.’ *Ibid*., p. 132. Emphasis original.

36 *Ibid*., p. 130. Emphasis original.

37 *Ibid*., pp. 196,197.

38 *Ibid*., p. 132,133.

39 *Ibid*., p. 468. Gross’ graphic approach has affinities with those being developed in contemporaneous field sciences. Gavin Lucas, for example, notes the shift in late nineteenth-century archaeological manuals away from artefact-based illustration to graphic representations of the site as a space of systematic excavation and investigation. Robert Kolher’s discussion of the introduction of the quadrat into ecological surveys is also relevant: the quadrat similarly functioned as a means of disciplining the surveyor by circumventing the human tendency to focus on (and thus to over-estimate the numbers of) ‘attractive’ plants. Lucas (2001), pp. 23,24, Kohler (2002), chapter 4, esp. pp. 100–108.

40 In this sense it again echoes developments in contemporaneous field sciences, most notably archaeology, which emphasised the need for producing a total record of a site irrespective of any prior hierarchy of value assigned to the objects that lay within any field. As discussed elsewhere, this sensibility is critical to the conceptualisation and operationalising of the early twentieth-century crime scene. Burney (2013).

41 Gross (1906), p. 642.

42 *Ibid*., p. 643.

43 *Ibid*.

44 *Ibid*. Emphasis original.

45 *Ibid*., p. 59.

46 The history of forensic photography has been dominated by accounts of Bertillon’s application of photography to criminal identification and its role and integration into his larger bureaucratic-statistical apparatus, which note the way in which the camera represented only one part of the archival practices underpinning his system of anthropometric identification. See, e.g., Sekula (1986), Thomas (1999), and Tucker (2005). Less systematic attention has been paid to Bertillon’s extension of this methodology to his approach to crime scene photography, which was designed to situate persons and objects of evidence within a metric screen that preserved critical information about the contours of the space (depth, scale) and its contents. For suggestive insights, see Becker (2001), pp. 213–214, Valier (1998), pp. 96–100, and most recently Castro (2011).

47 Gross (1906), p. 249.

48 *Ibid*.

49 *Ibid*., p. 252.

50 *Ibid*., p. 251.

51 *Ibid*., p. 255.

52 *Ibid*., pp. 264,265.
